# A systematic review of tools for predicting severe adverse events following patient discharge from intensive care units

**DOI:** 10.1186/cc12747

**Published:** 2013-06-29

**Authors:** F Shaun Hosein, Niklas Bobrovitz, Simon Berthelot, David Zygun, William A Ghali, Henry T Stelfox

**Affiliations:** 1 Medical Science Graduate Program, University of Calgary, 3330 Hospital Drive NW, Calgary, AB T2N 4N1, Canada; 2 Department of Community Health Sciences, Institute for Public Health, University of Calgary, Faculty of Medicine - 3rd Floor TRW Building, 3280 Hospital Dr. NW, Calgary, AB T2N 4Z6, Canada; 3 Department of Critical Care Medicine, University of Calgary, Ground Floor, McCaig Tower, 3134 Hospital Dr. NW, Calgary, AB T2N 2T9, Canada; 4 Department of Medicine, University of Calgary, Foothills Hospital, 1403 29th Street NW, Calgary, AB T2N 2T9, Canada

**Keywords:** Review, systematic, Critical care, Intensive care, Patient discharge, Decision support techniques, patient readmission, Mortality

## Abstract

**Introduction:**

The discharge of patients from the intensive care unit (ICU) to a hospital ward is a common transition of care that is associated with error and adverse events. Risk stratification tools may help identify high-risk patients for targeted interventions, but it is unclear if proper tools have been developed.

**Methods:**

We searched Ovid EMBASE, Ovid MEDLINE, CINAHL, PUBMED and Cochrane Central Register of Controlled Trials from the earliest available date through March 2013, plus reference lists and citations of all studies included in the systematic review. Cohort studies were selected that described the derivation, validation or clinical impact of tools for predicting medical emergency team activation, ICU readmission or mortality following patient discharge from the ICU. Data were extracted on the study design, setting, population, sample size, tool (components, measurement properties) and outcomes.

**Results:**

The literature search identified 9,926 citations, of which eight studies describing eight tools met the inclusion criteria. Reported outcomes included ICU readmission (*n = *4 studies), hospital mortality (*n = *3 studies) and both ICU readmission and hospital mortality (*n = *1 studies). Seven of the tools were comprised of distinct measurable component variables, while one tool used subjective scoring of patient risk by intensive care physicians. The areas under receiver operator curves were reported for all studies and ranged from 0.66 to 0.92. A single study provided a direct comparative analysis between two tools. We did not find any studies evaluating the impact of risk prediction on processes and outcomes of care.

**Conclusions:**

Eight risk stratification tools for predicting severe adverse events following patient discharge from ICU have been developed, but have undergone limited comparative evaluation. Although risk stratification tools may help clinician decision-making, further evaluation of the existing tools' effects on care is required prior to clinical implementation.

## Introduction

Transitions of patient care between providers have been identified as important routine processes of care that expose patients to preventable medical errors and adverse events [1]. The discharge of patients from the intensive care unit (ICU) to the hospital ward is one of the most challenging and high-risk transitions of care during which the sickest patients in the hospital change their provider team (physicians, nurses, pharmacists, therapists) and are transferred from a resource intensive environment to a resource-limited environment [2].

Determining when patients are ready for ICU discharge has traditionally been dependent on the clinical judgment of the physician discharging the patient from the ICU and the physician admitting the patient to the hospital ward. However, there has been increasing interest in the development of tools to describe patient risk at the time of discharge from ICU. Independent risk stratification may provide clinicians with additional information to guide clinical decision-making. Risk assessment may help target the delivery of resource-intensive transitional care interventions (for example, medical emergency teams) to patients at greatest risk. Intensive care unit readmission is increasingly used as a performance metric by many institutions and risk stratification models could be used to help benchmark such activities [3]. However, there is no consensus on an ICU discharge risk stratification tool.

Therefore, we performed a systematic review to synthesize the published literature on tools for predicting severe adverse events following patient discharge from ICU, describe their operating characteristics, and appraise their clinical effectiveness.

## Materials and methods

We searched for studies that evaluated tools to stratify patient risk of severe adverse events following ICU discharge. We used the Preferred Reporting Items for Systematic Reviews and Meta-Analysis (PRISMA) guidelines for conducting and reporting this systematic review [4].

### Data sources and search strategy

We conducted a systematic search of articles in Ovid EMBASE, Ovid MEDLINE, CINAHL, PUBMED and Cochrane Central Register of Controlled Trials from inception to March 2013. Searches were performed without year or language restrictions, and used combinations of the following three groups of terms: intensive care unit, patient discharge and severe adverse event (medical emergency team (MET) activation or ICU readmission or mortality). The search strategy for the MEDLINE database is depicted in Appendix A (see Additional file 1). We also searched references in the bibliographies of retrieved articles and performed a citation search of all studies included in the systematic review (that is, articles citing studies included in the systematic review). Search strategies were constructed with the help of an experienced information scientist (DL), and all citations were imported to an electronic database (Endnote X3, Thomson Reuters, New York, NY, USA).

### Article selection

Two authors independently reviewed titles and abstracts (SH, SB) for all studies identified in the search, followed by full text review of articles (SH, NB), identified by either reviewer as meeting inclusion criteria. Discrepancies were resolved by discussion between the reviewers. Kappa values and their 95% confidence intervals were calculated for agreement between authors.

We selected all articles that described the derivation, validation or evaluation of the clinical impact of a tool to stratify the patients' risk of adverse events following discharge from ICU. We defined severe adverse event as any one of the following: MET activation, ICU readmission, or death during a patient's hospital stay following discharge from ICU. MET activation was defined as emergency activation of an on-call resuscitation team for hospitalized patients meeting specific physiological criteria (as defined by individual hospital policy) following discharge from ICU.

Studies had to meet each of the following inclusion criteria: 1) the study described original research published in a peer reviewed journal; 2) the study populations were adult patients (majority patients >16 years) discharged from ICU; 3) the study described derivation, validation or clinical impact of a tool to risk stratify patients at the time of ICU discharge; and 4) the study reported at least one of the following three patient outcomes following patient discharge from ICU, MET activation, readmission to ICU or mortality. Studies not satisfying all four inclusion criteria were excluded from the review. We included only cohort studies and controlled trials. Case-control studies, case series and case reports were excluded. Systematic reviews were excluded, but their reference lists were hand-searched for relevant articles. Studies examining discharge from a high dependency or step-down unit were excluded.

### Data extraction

Two reviewers independently performed data abstraction and quality assessments. Reviewer consensus was required for inclusion of results. We extracted data describing study purpose, design, setting (country, type of ICU), sample size, study population (age, sex, illness severity), risk stratification tool (components, measurement properties) and outcomes (MET activation, ICU readmission, hospital mortality). Study quality was evaluated using pre-specified criteria (study design, patient follow-up, ethics approval, description of study patient eligibility criteria and characteristics, method of statistical adjustment, *a priori *consideration of sample size and power, study duration and discussion of limitations). Authors were contacted to request missing data.

### Data synthesis

We analyzed the abstracted data according to validated guidelines for narrative synthesis [5-8]. Studies were grouped according to outcomes measured. We compared each tool according to: model derivation and validation, operating characteristics and individual components/variables. Sensitivity, specificity and likelihood ratios were calculated using data presented in the article if not reported. Pooling of quantitative data was not possible due to the limited number of evaluations of individual risk stratification tools.

## Results

**Figure 1:**
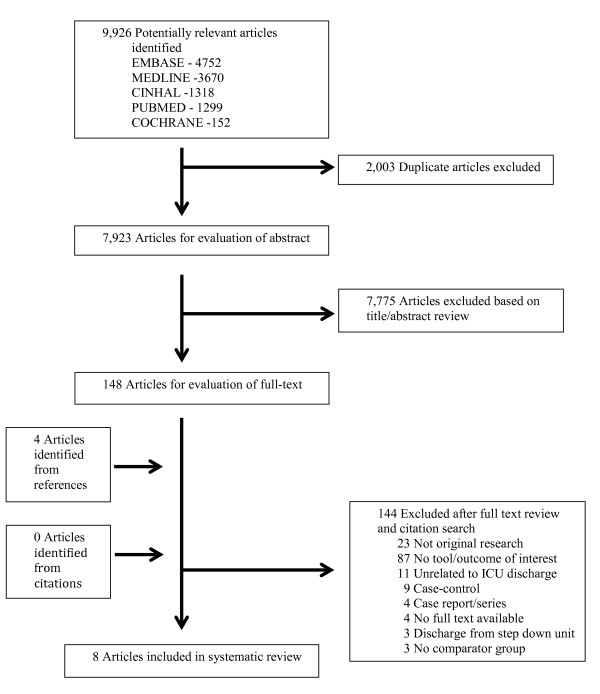
Selection of articles for review

Figure 1 describes the results of the article screening and selection process. The literature search identified 9,926 potentially relevant articles in five databases; from these we reviewed 148 full text articles and selected 8 articles for final inclusion in the study [9-16]. The two most common reasons for exclusion of articles after full-text review were that articles did not report original research or did not report study outcomes (MET activation, ICU readmission, hospital mortality). Inter-rater agreement was good for full-text review (kappa = 0.84, 95% confidence interval 0.67 to 1.00).

### Article characteristics and quality

Table 1 summarizes the characteristics of the articles included. Eight articles published between 2001 and 2012 described eight unique risk stratification tools. The studies were conducted primarily in medical-surgical ICUs in the United States and Europe. The number of patients in each study ranged from 518 to 704,963, with a total of 745,187 patients included in the review. The mean age of participants ranged from 57 to 64 years among the studies that reported age. Acute Physiology and Chronic Health Evaluation (APACHE) scores were used to measure patient severity of illness in the majority of studies. Five studies followed patients until hospital discharge. Among the included studies, following ICU discharge, ICU readmission ranged from 2.1% to 8.3% and hospital mortality from 0.4% to 9.6%. None of the studies reported MET activation.

**Table 1:** Characteristics of included studies

Study	Year	Country	Follow-up	Type of ICU	# Patients	Age (Mean)	Female (%)	Severity of Illness score (Mean)	Readmission **No**. (%)^ε^	Mortality No. (%)^ζ^
**Gajic**	2008	USA, Netherlands	7 days	Medical-Surgical	2,622	64^µ^	46^µ^	APACHE III (59)^µ^	217 (8.3)	5 (0.4)^µ^
**Frost**	2010	Australia	Hospital discharge	Medical-Surgical	14,952	57	39	APACHE II (13)	896 (6.0)	869 (6.0)
**Reini**	2012	Sweden	Hospital discharge	Medical-Surgical	518	59	46	SAPS III (55)	13 (3.7)	29 (8.8)
**Badawi**	2012	USA	48 hours	Mixed^§^	704,963	62	46	APACHE IV (47^β^)	17,874 (2.5)	6,492 (0.9)
**Daly**	2001	UK	Hospital discharge	Medical-Surgical	13,924	67-72^γ^	36	APACHE II (13)	142 (2.6)^µ^	1,158 (8.3)
**Fernandez**	2006	Spain	Hospital discharge	Medical-Surgical	1,159	60	N/A	APACHE II (20)^†^	N/A	111 (9.6)
**Fernandez**	2010	Spain	Hospital discharge	Medical-Surgical	3,587	61	33	N/A	190 (5.3)	242 (6.7)^α^
**Ouanes**	2012	France	7 days	Medical-Surgical	3,462	61	38	SAPS II (35)	74 (2.1)	28 (0.8)

APACHE, Acute Physiology and Chronic Health Evaluation; ICU, Intensive Care unit; N/A, Not Available; SAPS, Simplified Acute Physiology Score; UK, United Kingdom; USA, United States of America.

ε Readmission to ICU following patient discharge from ICU.

ζ Hospital mortality following patient discharge form ICU.

µ Data from derivation cohort.

§ Mixed ICUs: Medical, Medical-Surgical, Surgical, Trauma, Neurological, Cardiac, Cardiovascular.

β Median value.

γ Range of median ages for the derivation and validation cohorts.

† Point score estimated from predicted risk of death (percentage).

α Sensitivity analyses restricted to unexpected deaths produced the same mortality percentage.

### Description of risk stratification tools

Table 2 describes the eight risk stratification tools identified. The Sabadell score and the tool developed by Daly *et al*. were designed to predict hospital mortality following ICU discharge. Reini *et al*. evaluated the ability of the Modified Early Warning Score (MEWS) to predict ICU readmission within 72 hours of discharge. The Stability and Workload Index for Transfer (SWIFT) score and the Frost nomogram were developed to predict ICU readmission following ICU discharge. The Minimizing ICU Readmission (MIR) score was designed to predict the combined outcome of patient death or ICU readmission seven days post-ICU discharge. Badawi and Breslow developed two tools to respectively predict readmission and mortality 48 hours post-ICU discharge. All tools except the Sabadell score incorporated between 5 and 26 variables into their risk calculation with length of ICU stay the only common variable appearing in most tools. The Sabadell score was calculated by physician judgment of patient prognosis at the time of ICU discharge using a four-point scale.

**Table 2:** Risk stratification tools

**Author**, Tool, Country Year	Tool Components/ Variables (Weighting/Points)	Prediction Outcome (Follow-up)	Tool Development (# patients)	Tool Validation (# patients/#ICU)	Sensitivity (%)	Specificity (%)	LR+	LR-	AUROC (95% CI)
**Gajic** **'SWIFT Score'** **USA, 2008**	Source of ICU admission (Other than ED: 8 pt) ICU length of stay (2 to 10 d: 1 pt, >10 d: 4 pt) Last measured PaO2/FiO2 ratio (150 to 399: 5 pt, 100 to 149: 10 pt, <100: 13 pt) GCS at ICU discharge (11 to 14: 6 pt, 8 to 10: 14 pt, <8: 24 pt) Last PaCO2 (>45 mmHg: 5 pt)	Readmission (7 days)	Multivariate (1,131)	Internal (783/1) External (708/1)	56 27	83 87	3.09 2.13	0.56 0.84	0.75 (0.70 to 0.80) 0.70 (0.64 to 0.76)
**Frost** **Australia, 2010**	Age (years: 0 to 8 pt) Male (2 pt) Elective admission (12 pt) Admission source (ED: 9 pt, Other hospital: 10 pt, Ward: 15 pt) APACHE II score (0 to 20 pt) ICU length of stay >7 days (17 pt) After hours discharge (4 pt) Renal failure (10 pt)	Readmission (Hospital Discharge)	Multivariate (14,952)	Internal^‡^ (14,952/1)	n/a	n/a	n/a	n/a	0.66 (n/a)
**Reini** **Sweden, 2012**	Pulse rate (0 to 3 pt) Respiratory rate (0 to 3 pt) Systolic blood pressure (0 to 3 pt) Level of consciousness (0 to 3 pt) Temperature (0 to 2 pt)	Readmission (72 hours)	Existing Score	External (518)	15^δ^	85^δ^	1.01^δ^	0.99^δ^	OR 0.98 (0.69 to 1.37)^¶^
**Badawi** **USA, 2012**	23 variables^λ^	Readmission (48 hours)	Multivariate (469,967)	Internal (234,976/219)	6 to 96^ψ^	19 to 99^ψ^	1.19 to 5.72^ψ^	0.19 to 0.95^ψ^	0.71 (0.71 to 0.71)
	26 variables^§^	Mortality (48 hours)	Multivariate (469,967)	Internal (234,976/219)	47 to 82^ψ^	87 to 99^ψ^	6.44 to 55^ψ^	0.20 to 0.53^ψ^	0.92 (0.92 to 0.92)
**Daly** **United Kingdom**, **2002**	β coefficients^†^ Age per year (0.0532) Chronic Health Points (0.2501) ICU length of stay per day (0.0447) Acute Physiology points (0.1556) Cardiothoracic surgery (-2.104) Constant (-4.5821)	Mortality (Hospital Discharge)	Multivariate (5,475)	Internal (1,136/1) External (7,313/19)	74	71	2.55	0.37	0.80 (0.79 to 0.81)^θ^
**Fernandez** **'Sabadell Score' Spain**, **2006 and 2010**	Subjective intensive care physician scoring: Good Prognosis (0) Poor long term prognosis, >6 months (1) Poor short term prognosis, <6 months (2) Death expected within hospitalization (3)	Mortality (Hospital Discharge)	Existing Score Modified	Internal (1,521/1) External (3,587/31)	23 to 87 26 to 85	79 to 99 71 to 99	4.14 to 23 2.93 to 26	0.16 to 0.78 0.21 to 0.75	0.88 (0.84 to 0.93) 0.84 (0.81 to 0.87)
**Ouanes** **'MIR'** **France**, **2012**	β coefficients^†^ SAPS II (admission) (0.017) Central venous catheter (0.74) SIRS (max) (0.61) SOFA (discharge) (0.19) Discharge at night (0.92) Constant (-5.59)	Readmission or mortality (7 days)	Multivariate (3,462)	Internal^‡^ (3,462/4)	50 to 96^α^	19 to 82^α^	1.19 to 2.78^α^	0.21 to 0.61^α^	0.74 (0.68 to 0.79)

APACHE, Acute Physiology and Chronic Health Evaluation; AUROC, area under the receiver operating characteristic curves; ED, emergency department; GCS, Glasgow Coma Scale; ICU, Intensive Care unit; LR, likelihood ratio; MIR, Minimizing ICU Readmission; N/A, Not Available; SAPS, Simplified Acute Physiology Score; SIRS, Systemic Inflammatory Response Syndrome; SOFA, Sequential Organ Failure Assessment score; SWIFT, Stability and Workload Index for Transfer; USA, United States of America.

‡ Resampling using bootstrap techniques.

δ Calculated using the Modified Early Warning Score on admission to ICU of <6 vs. >6.

¶ Odds ratio for readmission to ICU within 72 hours of ICU discharge reported for each one point increase in the Modified Early Warning Score at the time of ICU discharge. The receiver operating characteristic curve not reported.

λ Readmission model variables: Admission characteristics (age), Elective surgery, ICU type, Admission diagnosis category, Admit source, ICU visit number, Body mass index, ICU interventions (number of lactate values in 24 hours, ICU length of stay), Last day labs, (serum bicarbonate, white blood cell count, serum creatinine, hemoglobin), Last day physiology (heart rate, respiratory rate, diastolic blood pressure, systolic blood pressure, percent oxygen, most recent Glasgow coma scale score).

ψ Range of sensitivities and specificities reported for four different thresholds of predicted readmission or mortality following ICU discharge ranging from 1% to 10%.

§ Mortality model variables: Admission characteristics (age, body mass index), Operative diagnosis (elective surgery), ICU interventions (ICU length of stay, ventilation status), Last day labs (serum lactate, serum creatinine, white blood cell count, serum glucose), Last day physiology (diastolic blood pressure, heart rate, mean arterial pressure, respiratory rate, percent oxygen saturation, most recent Glasgow coma scale score).

† β coefficients reported from multivariable regression model.

θ AUROC reported for combined data for internal and external validation cohorts.

α Range of sensitivities, specificities and likelihood ratios reported for four different thresholds of the MIR score.

Evaluations of internal and external validity were reported for most tools. The calculated area under the receiver operating characteristic curves (AUROC) ranged from 0.66 to 0.92, with the Badawi and Breslow mortality tool having the highest reported AUROC (0.92). The sensitivity, specificity and likelihood ratios were reported or could be calculated for all the tools except the Frost nomogram. Gajic *et al*. compared the SWIFT score to the APACHE III score (AUROC 0.75 vs. 0.62, *P *<0.01) at the time of patient discharge from ICU. Ouanes *et al*. similarly compared the MIR score, SWIFT score and Simplified Acute Physiology Score II (AUROC 0.74 vs. 0.61 vs. 0.64).

We did not find any studies evaluating the impact of risk prediction on processes and outcomes of care.

### Study quality

Table 3 summarizes study quality. All studies used a cohort design (four prospective, four retrospective), described patient eligibility criteria, reported complete patient follow-up and performed multivariate data analysis. The studies by Badawi and Breslow, Daly *et al*., Fernandez *et al*. and Ouanes *et al*. include more than one ICU. Half of the studies included patients with do-not-resuscitate orders at the time of ICU discharge.

**Table 3:** Description of study quality

Study	Year	Cohort timing	ICU #	Ethics approval reported	Follow-up complete	Demographics described	DNR Patients included	Comorbidities assessed	Type of analysis	SOI score used	Eligibility criteria mentioned	Power calculated *a priori*	Study duration justified	Limitations discussed
**Gajic**	2008	Prospective	1	Yes	Yes	Yes	No	No	Multivariate	Yes	Yes	No	No	Yes
**Frost**	2010	Retrospective	1	Yes	Yes	Yes	No	Yes	Multivariate	Yes	Yes	No	Yes	Yes
**Reini**	2012	Prospective	1	Yes	Yes	Yes	Yes	No	Multivariate	Yes	Yes	No	Yes	Yes
**Badawi**	2012	Retrospective	219	Yes	Yes	Yes	No	No	Multivariate	Yes	Yes	No	Yes	Yes
**Daly**	2001	Retrospective	20	Yes	Yes	Yes	Yes	No	Multivariate	Yes	Yes	No	No	No
**Fernandez**	2006	Prospective	1	No	Yes	No	Yes	Yes	Multivariate	Yes	Yes	No	Yes	Yes
**Fernandez**	2010	Prospective	31	No	Yes	Yes	Yes	Yes	Multivariate	Yes	Yes	No	Yes	Yes
**Ouanes**	2011	Retrospective	4	Yes	Yes	Yes	No	Yes	Multivariate	Yes	Yes	No	Yes	Yes

DNR, Do Not Resuscitate; ICU, Intensive Care unit; SOI, Severity of Illness

## Discussion

Our systematic review identified eight ICU discharge risk stratification tools evaluated in eight studies. Outcome parameters from these studies were ICU readmission, post-ICU mortality and a combination of both. All tools except the Sabadell score used patient physiological and clinical characteristics to calculate patient risk of severe adverse events following ICU discharge. The SWIFT score, Badawi and Breslow mortality tool and MIR score had the best reported operating characteristics for predicting ICU readmission, hospital mortality and the combined outcome of ICU readmission and hospital mortality, respectively. A single study compared two of the risk stratification tools. No studies reported MET activation following ICU discharge as an outcome, identifying an opportunity for evaluation in future studies.

Our study adds to the literature by highlighting an important gap in the science of patient care transitions from the ICU. First, it is unclear whether a reliable and valid risk stratification tool for patient discharge from ICU has been developed. We identified eight risk stratification tools, only two of which have been directly compared in a single study, where the MIR score had a better AUROC value than the SWIFT, although lower than those reported for the tools developed by Daly *et al*., Fernandez *et al*. and Badawi and Breslow. Furthermore, only the MIR score is designed to predict both post-ICU discharge mortality and readmission. To complicate matters it is unclear which of these two outcomes is most relevant. These data suggest that the MIR score is promising, although its use of beta coefficients to calculate patient risk will make it difficult for clinicians to use outside of a computerized algorithm. The ICU discharge readiness scores developed by Badawi and Breslow have not been compared to other tools, and their proprietary nature may make independent evaluation challenging, but their derivation and evaluation in a large number of patients and ICUs suggests promise.

Second, it is also unclear how risk stratification tools compare to physician clinical judgment for identifying patients at increased risk of adverse events post-ICU. Clinical estimation is often based on subjective parameters and physicians can be overconfident in their predictive abilities [17-20]. Independent risk stratification may provide clinicians with additional information to guide clinical decision-making, but further evaluation is required. To complicate matters, patient populations can vary substantially between ICUs (for example, multisystem ICUs vs. subspecialty ICUs) suggesting that development of a tool that can be broadly applied across patient populations may be difficult [21].

Third, it is unknown whether implementation of a risk stratification tool can improve processes and outcomes of care for patients. Laupacis *et al*. outlined three evaluation criteria for clinical prediction rules [22]. One, the tool needs to be validated to provide evidence of reproducible accuracy. Two, the tool needs to have sufficient predictive power to provide clinicians with confidence to use the results to guide decision-making. Three, the tool needs to be actually used by clinicians (easy to use whether using memory, paper-based tool or electronic tool) to change behavior and improve patient outcomes. Ideally, an ICU discharge risk stratification tool would forecast patient outcomes and, therefore, facilitate the delivery of safe (for example, reduce premature ICU discharge for high risk patients), effective (for example, target transition resources to high risk patients) and efficient (for example, expedite ICU discharge for low risk patients) care. The first two evaluation criteria are satisfied to varying degrees by the tools identified in our review. However, prior to implementation, an impact analysis demonstrating evidence that risk stratification changes physician behavior and improves patient outcomes is needed [22].

Our study has limitations. First, relevant studies may have been missed despite using comprehensive search strategies and the assistance of an information specialist to search multiple databases. Second, our study was limited to reviewing studies of existing risk stratification tools. We are, therefore, unable to comment on studies that did not include tools but that may contain evidence that warrants the development of new tools. Third, we identified a small number of studies with heterogeneous populations that measured different outcomes, which limited direct comparison of risk stratification tools.

## Conclusion

In summary, risk stratification of patients discharged from ICU is a complex process with many potential challenges. Several risk stratification tools have been developed with the MIR score and the Badawi and Breslow mortality tool appearing to be promising. However, at present it is unclear whether existing tools provide value above clinician judgment, or whether they can be used to improve healthcare delivery. Further evaluation of existing tools is required prior to clinical implementation.

## Key messages

• Transition of patient care from ICU is a high-risk and challenging transition that is associated with severe adverse events.

• Tools to stratify patient risk of readmission or death following discharge from ICU have been developed, but are not ready for clinical application.

• Additional comparative evaluations of tool reproducible accuracy are needed as well as impact analyses demonstrating evidence that risk stratification changes physician behavior and improves patient outcomes.

## Abbreviations

APACHE: Acute Physiology and Chronic Health Evaluation; AUROC: area under the receiver operating characteristic curve; ICU: intensive care unit; MET: medical emergency team; MEWS: Modified Early Warning Score; MIR: Minimizing ICU Readmission; PRISMA: Preferred Reporting Items for Systematic Reviews and Meta-analysis; SAPS: Simplified Acute Physiology Score; SWIFT: Stability and Workload Index for Transfer

## Competing interests

The authors declare that they have no competing interests.

## Authors' contributions

All six authors contributed to the study's conception, design and interpretation. FSH and SB were responsible for searching the literature and reviewing abstracts. FSH and NB were responsible for selecting manuscripts and critically appraising them. FSH, NB, SB and HTS performed the analyses. All six authors assisted in the successive revisions of the final manuscript. All authors have read and approved the final manuscript.

## Supplementary Material

Additional file 1 **Appendix A**. MEDLINE Search Strategy. The list of terms used to conduct a systematic search of Ovid EMBASE, Ovid MEDLINE, CINAHL, PUBMED and Cochrane Central Register of Controlled Trials. Click here for file
