# Supplementary material for: A systematic review of tools for predicting severe adverse events following patient discharge from intensive care units
Source: Crit Care. 2013 Jun 29;17(3):R102. doi: 10.1186/cc12747 (PMC4056089; doi:10.1186/cc12747)
Supplement: Additional file 1 — Appendix A. MEDLINE Search Strategy. The list of terms used to conduct a systematic search of Ovid EMBASE, Ovid MEDLINE, CINAHL, PUBMED and Cochrane Central Register of Controlled Trials. [file cc12747-S1.DOC]

**Appendix A. MEDLINE Search Strategy**

1. exp Intensive Care Units/

2. exp Intensive Care/ or exp Critical Care/

3. exp Critical Illness/

4. special care unit.tw.

5. close attention unit.tw.

6. intensive care department.tw.

7. intensive care units.tw.

8. respiratory care unit.tw.

9. intensive care.tw.

10. critical care.tw.

11. icu.tw.

12. icu.tw.

13. exp Patient Discharge/

14. exp Patient Transfer/

15. (patient adj5 discharg*).tw.

16. (discharg* adj5 planning).tw.

17. patient dumping.tw.

18. intrahospital transfer.tw.

19. discharge.tw.

20. exp Patient Readmission/

21. exp Patient Readmission/

22. Readmission.tw.

23. (Patient adj5 readm*).tw.

24. (Unit adj8 readm*).tw.

25. (Unplan* adj5 adm*).tw.

26. exp Hospital Mortality/ or exp Mortality/

27. death rate*.tw.

28. mortality determinant.tw.

29. differential mortalit*.tw.

30. age-specific death rates.tw.

31. mortality decline.tw.

32. premature mortality.tw.

33. case fatality rate.tw.

34. in-hospital mortal*.tw.

35. inhospital mortal*.tw.

36. hospital mortal*.tw.

37. exp Death, Sudden/

38. exp Death, Sudden, Cardiac/

39. exp Hospital Rapid Response Team/

40. rapid response team.tw.

41. cardiac crash team.tw.

42. code team*.tw.

43. team code.tw.

44. medical emergency team.tw.

45. medical care team.tw.

46. health care team.tw.

47. healthcare team.tw.

48. exp Cardiopulmonary Resuscitation/

49. Code blue.tw.

50. 1 or 2 or 3 or 4 or 5 or 6 or 7 or 8 or 9 or 10 or 11 or 12

51. 13 or 14 or 15 or 16 or 17 or 18 or 19

52. (unplan* adj5 admi*).tw.

53. 21 or 22 or 23 or 24 or 25 or 26 or 27 or 28 or 29 or 30 or 31 or 32 or 33 or 34 or 35 or 36 or 37 or 38 or 39 or 40 or 41 or 42 or 43 or 44 or 45 or 46 or 48 or 49 or 52

54. 50 and 51 and 5
